# Discovery, Identification, and Insecticidal Activity of an *Aspergillus flavus* Strain Isolated from a Saline–Alkali Soil Sample

**DOI:** 10.3390/microorganisms11112788

**Published:** 2023-11-16

**Authors:** Yuxin Song, Xiaoli Liu, Shirong Feng, Kangbo Zhao, Zhijun Qi, Wenjun Wu, Jie Xiao, Hong Xu, Mingwei Ran, Baofu Qin

**Affiliations:** 1 College of Life Sciences, Northwest A&F University, Xianyang 712100, China; syx1172598498@163.com (Y.S.); l3136014743@163.com (X.L.); fengshirong@nwafu.edu.cn (S.F.); emmayaoshangan@163.com (K.Z.); aqiu829@nwafu.edu.cn (J.X.); xuh73@163.com (H.X.); 17703228112@nwafu.edu.cn (M.R.); 2 College of Plant Protection, Northwest A&F University, Xianyang 712100, China; qzhij@nwsuaf.edu.cn (Z.Q.); wuwenjun@nwsuaf.end.cn (W.W.); 3 Institute of Pesticides, Northwest A&F University, Xianyang 712100, China

**Keywords:** biological control, *Aspergillus flavus*, fungal spores, aphids, enzyme activity, histopathology

## Abstract

Aphids are one of the most destructive pests in agricultural production. In addition, aphids are able to easily develop resistance to chemical insecticides due to their rapid reproduction and short generation periods. To explore an effective and environmentally friendly aphid control strategy, we isolated and examined a fungus with aphid-parasitizing activity. The strain (YJNfs21.11) was identified as *Aspergillus flavus* by ITS, 28S, and *BenA* gene sequence analysis. Scanning electron microscopy and transmission electron microscopy revealed that the infection hyphae of ‘YJNfs21.11’ colonized and penetrated the aphid epidermal layer and subsequently colonized the body cavity. Field experiments showed that ‘YJNfs21.11’ and its fermentation products exerted considerable control on aphids, with a corrected efficacy of 96.87%. The lipase, protease, and chitinase secreted by fungi help aphid cuticle degradation, thus assisting spores in completing the infection process. Additionally, changes were observed in the mobility and physical signs of aphids, with death occurring within 60 h of infection. Our results demonstrate that *A. flavus* ‘YJNfs21.11’ exhibits considerable control on *Aphis gossypii* Glover and *Hyalopterus arundimis* Fabricius, making it a suitable biological control agent.

## 1. Introduction

Aphids are highly diverse, with 10 families and 4400 species discovered to date [1]. Unfortunately, aphids are also one of the most destructive crop pests, reducing both quality and yield in diverse plants, including wheat [2], melons [3,4], peaches [5,6], and cotton [7], among others. Currently, chemical pesticide application is considered the most efficacious method of control. However, chemical pesticides alter the molecular physiology of target insects, resulting in altered enzyme activity, genetic mutations, and pesticide resistance [8]. In addition, chemical pesticides have been linked to both environmental and food safety risks.

To address these issues, the importance of entomopathogenic fungi as alternative pest control agents is increasing. For example, certain fungi have been found to effectively control aphids [9]. These include *Beauveria bassiana* [10,11,12], the *Metarhizium* [13,14] and *Verticillium* [15,16] genera, as well as fungi belonging to the Entomophthorales order [17]. These mycopesticides mainly use propagules such as conidia, blastospores, or hyphae. These propagules have the advantages of directly killing the target pest as well as secondary infection via horizontal transmission of spores from cadavers with mycosis. Several of these fungi have been used to develop wettable powder-based biopesticides for use against foliar sap-sucking pests [18].

Based on initial reports, the cosmopolitan saprophytic fungus *Aspergillus flavus* may be a promising aphid biocontrol agent [19,20]. Laboratory bioassays indicate that *A. flavus* is pathogenic to aphids at doses of 1.23 × 10^3^ spores/mL (LC50) and 1.34 × 10^7^ spores/mL (LC90) [21]. In another study involving cabbage and wheat, 33% of cabbage aphids and 37% of wheat aphids were found to be susceptible to fungal biocontrol, with *A. flavus* among the most effective pathogens [22]. However, the mechanism by which *A. flavus* exerts control over aphids, and the active substances involved, remains largely unknown.

In this study, we isolated *A. flavus* from a sample of Chinese saline–alkali soil. The insecticidal activity of the strain was verified with lab-based and field-based bioassays. Subsequently, microscopic techniques were used to analyze histopathological changes in infected aphids and the influence of enzyme activity on biocontrol efficacy was explored. The results presented here will be valuable for the realization of sustainable aphid control strategies.

## 2. Experimental Procedures

### 2.1. Isolation and Identification of the Strain

In October 2021, we isolated *a* strain in saline–alkali soil sample from Ningxia Autonomous Region, China. The soil sample was first cleared of debris and filtered using a 100-mesh screen, then washed and rinsed three times with sterile water [23]. Subsequently, the soil was diluted 10×, 10^2^×, 10^3^×, 10^4^×, 10^5^×, 10^6^×, 10^7^×, and 10^8^× with sterile water and inoculated (100 µL) onto potato dextrose agar (PDA) using the spread plate technique [24,25]. Each gradient was repeated three times and was cultured for 3–5 d in a constant temperature (28 °C) incubator. Purification was repeated until a single colony was obtained [26].

The strain was identified based on ITS, LSU (28S), and *BenA* gene sequence (Supplementary Materials) analysis. Briefly, genomic DNA was isolated with a DNA extraction kit (Solarbio Science & Technology Co., Ltd., Beijing, China) and used as the PCR amplification template. The forward primer and reverse primer were synthesized by Beijing Qingke Biotechnology (Beijing, China). The amplified products were subjected to Sanger two-way sequencing. The ITS gene was amplified using the ITS1 primer (5′-TCCGTAGGTGAACCTGCGG-3′) and ITS4 primer (5′-TCCTCCGCTTATTGATATGC-3′); the LSU (28S) gene was amplified using the LR0R (5′-GTACCCGCTGAACTTAAGC-3′) and LR12 (5′-GACTTAGAGGCGTTCAG) primers; and the *BenA* gene was amplified using the Bt2a (5′-GGTAACCAAATCGGTGCTGCTTTC-3′) and Bt2b (5′-ACCCTCAGTGTAGTGACCCTTGGC-3′) primers. A BLAST search (NCBI) was conducted and the strain was identified as the species sharing the highest homology (>97%). Colony morphology was evaluated using a stereo microscope (NIKON, Tokyo, Japan) and a tungsten filament scanning electron microscope (JSM-6360LV, Nippon Electronics, Tokyo, Japan).

### 2.2. Extraction and Separation of Active Substances and Preparation of Reagents

#### 2.2.1. Preparation of Spore Suspension

To prepare the spore suspension, incubation plates were washed with sterile water on a sterile operating table. The spores were collected in a conical flask with glass beads, shaken thoroughly, and scattered. A single spore suspension was obtained via filtration with a cotton-stuffed syringe. The concentration was adjusted to 1 × 10^7^ spores/mL using a hemocytometer. Finally, 0.1% tween-20 was added and the suspension was refrigerated (4 °C) for subsequent analyses [27,28].

#### 2.2.2. Preparation of Fermentation Solution

The strain was fermented under aerobic conditions. The fermentation medium (35 g soluble starch, 15 g sucrose, 12.5 g yeast extract, 7.5 g soybean cake powder, 1.0 g KH_2_PO_4_, 1.1 g anhydrous MgSO_4_, 1.0 g NaCl) was prepared in 1 L of distilled water and distributed into ten 250 mL conical flasks. The flasks were sealed with eight layers of gauze and then autoclaved for 20 min. The spore suspension was injected into the fermentation medium at a concentration of 5%. Following inoculation, the flasks were again sealed with eight layers of gauze and cultured for 3 d in a shaker (165 r/min) at 32 °C. Next, the fermentation solution was homogenized (JJ-2, Hangzhou, China) to prevent mycelium from clogging the nozzle. Finally, 0.1% tween-20 was added. We found that sealing with gauze was more beneficial to the growth of *A. flavus*. This may be due to the breathability of the gauze. In order to prevent bacterial contamination, we used gauze to seal the conical bottles and then coated the gauze in kraft paper to maintain sterility and prevent water vapor pollution. In addition, after inoculation we used ultraviolet radiation to sterilize the replacement gauze.

#### 2.2.3. Preparation of Mycelium Reagents 

The fermented broth was filtered with eight layers of gauze and eluted three times with water. Next, the mycelium was weighed, homogenized, and diluted 100×. Finally, 0.1% tween-20 was added and the suspension was refrigerated (4 °C) for subsequent analyses.

#### 2.2.4. Preparation of Methanol Extract Reagent

After the fermentation solution was filtered with eight layers of gauze, the mycelium was weighed. Next, the mycelium was mixed with methanol at a ratio of 1:10 and ultrasonicated for 30 min. This process was repeated twice, followed by dilution with 0.1% tween-20 to ensure that the organic solvent content was ≤1%. The resulting methanol extract reagent was refrigerated (4 °C) for subsequent analyses.

#### 2.2.5. Preparation of Fermentation Filtrate

After filtration with 8 layers of gauze, the fermentation solution was centrifuged (12,000 r/min) for 5 min and the supernatant was filtered using a 0.22 µm microporous filtration membrane. Finally, 0.1% tween-20 was added to the filtered supernatant and the suspension was refrigerated (4 °C) for subsequent analyses.

### 2.3. Evaluation of Biocontrol Efficacy

Here, we used naturally occurring *A. gossypii* and *H. arundimis* in order to better test the aphicidal activity of the strain. Naturally occurring aphid populations are more resilient and resistant to biotic and abiotic stressors than lab-reared aphids. In addition, due to exposure to environmental toxins and pesticides, naturally occurring aphids are much more resistant to biotic and abiotic stressors, and reproduce more quickly, than lab-reared aphids.

In the lab-based bioassay [29,30,31], we tested the efficacy of this strain against *H. arundimis*, which thrive on peach trees and are often difficult to control because of their thick waxy layers. Screening experiments were performed to measure the efficacy of the strain on aphid nymphs. All treatments were carried out in 90 mm Petri dishes. Peach leaves exhibiting vigorous growth and hosting large numbers of aphids were excised, and the petioles were wrapped in cotton moistened with water. The experimental leaves were immersed in both the original and diluted (2×, 10×, and 50×) fermentation broths for approximately 3 s [32], with 0.1% tween-20 used as a negative control, followed by blotting with absorbent paper to remove excess liquid. The aphids were counted and then transferred to sterile Petri dishes containing a 9 cm diameter section of filter paper. Three parallel experiments were carried out for each group. Dishes were placed in a culture chamber with a temperature of 25 (±1) °C and a relative humidity of ≥85% [33]. Aphid mortality rates were recorded at 16 h, 24 h, and 48 h after treatment. Subsequently, the population reduction rate and corrected efficacy were calculated according to the equations below.

In the field-based bioassay, we tested the aphicidal efficacy of this strain against *A. gossypii*, which are a major pest of fruit and vegetable crops such as melons. Both the original and diluted (2×, 10×, and 50×) fermentation broths were sprayed evenly on the leaves of vigorously growing muskmelon, with 0.1% tween-20 used as a negative control. Five to eight muskmelon plants were used for each treatment. Three parallel experiments were carried out for each group. The test area (1 m^2^) was randomly arranged in blocks, and no other chemical agents were applied to the field during the test [34]. The number of aphids was recorded before application, and the number of dead aphids was recorded on the 1st, 3rd, and 5th day after application. Subsequently, the population reduction rate and corrected efficacy were calculated according to the equations below. Corrected efficacy refers to a statistical method for correcting calculations of control efficacy over a specific treatment area to account for changes in insect body condition. The day of the experiment was sunny with a suitable temperature. The experiment was carried out in the key open dryland test field of Northwest A&F University, China. The experimental field contained loamy soil of medium fertility.

$$Population\;reduction\;rate\;(\%)=\frac{initial\;aphid\;count-aphid\;count\;after\;treatment}{initial\;aphid\;count}\times 100\%$$

$$Corrected\;efficacy\;(\%)=(1-\frac{initial\;control\;aphid\;count\times experimental\;aphid\;count\;after\;treatment}{initial\;experimental\;aphid\;count\times control\;aphid\;count\;after\;treatment})\times 100\%$$

### 2.4. Evaluation of Histopathologic Changes in Infected Aphids 

Subsequent to this strain exposure, the feeding ability, activity [35], and symptoms of infected and control *A. gossypii* were observed continuously with a stereomicroscope (LEICA M165 FC, LEICA, Wetzlar, Germany). At 24 h, 36 h, 48 h, and 60 h post infection, *A. gossypii* were fixed with 2.5% glutaraldehyde at 4 °C. Then, the levels of this strain adhesion, germination, and invasion were observed using scanning electron microscopy. In addition, at 24 h, 36 h, 48 h, and 60 h post infection, the susceptible *A. gossypii* were fixed for 3 h with paraformaldehyde at 4 °C. The fixed specimens were infiltrated and embedded in Epon 812, then sliced (80 µm thickness) using an ultrathin slicer (EMUC7, Leica Instrument Co. LTD, Vienna, Austria), fixed on a copper mesh, stained with uranium acetate and lead citrate, and observed using transmission electron microscopy (TECNAI G2 SPIRIT BIO, FEI Corporation, Hillsboro, OR, USA).

### 2.5. Evaluation of the Effect of the Strain Infection on Aphid Body Wall Extracellular Enzyme Degradation

#### 2.5.1. Formulation of Culture Medium from Aphid Cuticles

*H. arundimis* were carefully removed from peach tree branches with paintbrushes and placed in beakers, and then subsequently rinsed repeatedly with tap water to remove impurities. The internal organs of the aphids were removed using a dissecting microscope (SZ51, Beijing, China), and the wax shells and body walls of the aphids were dried in an oven at 65 °C. Next, the dried body walls were ground into powder and sieved (40 mesh). In total, 5 g of aphid body wall powder was added to the base medium (1.5 g KH_2_PO_4_, 0.6 g MgSO_4_, 0.5 g KCl, 1 mg FeSO_4_·7H_2_O, 1 mg ZnSO_4_·7H_2_O, 1 L distilled water). A total of 40 mL of the mixture was allocated to each 150 mL conical vial and autoclaved (120 °C for 30 min). This process ensured that the liquid medium contained aphid body walls and wax shells as the only carbon sources.

#### 2.5.2. Extraction of Crude Enzyme Solution

Each conical vial was inoculated with 10 mL of spore suspension and placed in a thermostatic shaking table (28 °C, 150 r/min) for 8 d. Three 0.5 mL samples were collected each day and centrifuged (10,000 r/min for 20 min, 4 °C). The supernatant was collected as the crude enzyme solution and stored in an ultralow temperature refrigerator at −80 °C for subsequent analyses.

#### 2.5.3. Determination of Lipase Activity

Briefly, 3 mg of p-nitrophenyl palmitate (p-NPP) was dissolved in 1 mL of isopropyl alcohol, and the mixture was subsequently dissolved in 9 mL of Tris-HCl (0.05 mol/L, pH 8.0) containing 20.7 mg sodium deoxycholate and 10 mg gum arabic to prepare the matrix solution. During the reaction, 200 μL of the substrate solution was preheated in a 37 °C water bath for 10 min, and then 20 μL of enzyme solution was added and stirred uniformly. Next, 300 μL of trichloroacetic acid was added after water bath heating (37 °C, 20 min) and stirred well, then left to stand at room temperature for 5 min to terminate the reaction. Finally, 320 μL of NaOH (0.05 mol/L) was added. For the control group, trichloroacetic acid was added first, followed by the enzyme solution. OD values (410 nm) were measured using a microplate reader (Bio Tek Synergy H1, Winooski, VT, USA). The enzyme activity was calculated according to the p-nitrophenol standard curve. A lipase activity unit was defined as the amount of enzyme catalyzing the decomposition of fat to produce 1 μg of p-nitroaniline per minute.

#### 2.5.4. Determination of Protease Activity

A 1% (*w*/*v*) casein solution was prepared with Tris-HCl buffer (0.05 mol/L, pH 8.5). In total, 1 mL of this solution was mixed with 1 mL of the prepared enzyme solution in a centrifuge tube and heated in a water bath (37 °C for 30 min). The reaction was terminated with 3 mL trichloroacetic acid (0.4 mol/L). For the control group, 1 mL of enzyme solution and 3 mL of trichloroacetic acid (0.4 mol/L) were successively added to the centrifuge tube, and then 1 mL of casein solution was added after mixing. Next, 1 mL of the filtrate was added to 5 mL of 0.55 mol/mL Na_2_CO_3_ and mixed well. Finally, 1 mL of Folin reagent was added and the solution was mixed well and reacted for 30 min. OD values (680 nm) were measured using a microplate reader. The enzyme activity was calculated according to the tyrosine standard curve. A protease activity unit was defined as the amount of enzyme catalyzing the decomposition of protein to produce 1 μg of tyrosine per minute.

#### 2.5.5. Determination of Chitinase Activity

Both 0.1 mL of enzyme solution and 0.1 mL of colloidal chitin were mixed in a centrifuge tube, water-bathed (37 °C for 4 h), and then centrifuged (8000 r/min for 5 min) to terminate the enzymatic hydrolysis reaction. A total of 50 μL of the supernatant was mixed with 20 μL of potassium tetraborate solution, shaken thoroughly, reacted in a boiling water bath for 5 min, and then quickly cooled to room temperature with tap water. Next, 300 μL of 10% dimethylaminobenzaldehyde reagent was added and the mixture was water-bathed (37 °C for 20 min) and subsequently cooled to room temperature with tap water. OD values (585 nm) were measured using a microplate reader. The enzyme activity was calculated according to the N-acetylglucosamine standard curve. A chitinase activity unit was defined as the amount of enzyme catalyzing the decomposition of chitin to produce 1 μg of N-acetylglucosamine per minute.

### 2.6. Statistical Analyses

In order to eliminate certain analytical errors, exact values were utilized for statistical analyses. SPSS 26.0 was used to perform single-factor analysis of variance, and the results were expressed as the mean ± standard deviation. Duncan’s method was used for multiple comparisons. Statistical significance was determined at the *p* < 0.05 level.

## 3. Results

### 3.1. Isolation and Identification of the Strain

**Figure 1:**
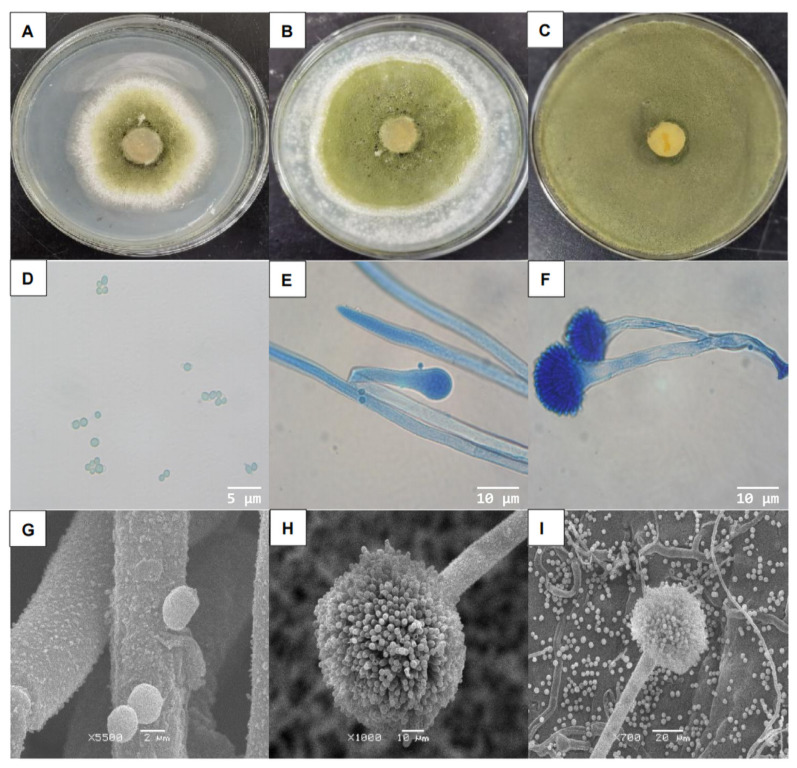
Colony morphology and microscopic examination of *A. flavus* ‘YJNfs21.11’. Note: (**A**) ‘YJNfs21.11’ colony morphology after 2 d of growth; (**B**) ‘YJNfs21.11’ colony morphology after 4 d of growth; (**C**) ‘YJNfs21.11’ colony morphology after 7 d of growth; (**D**) conidia of strain ‘YJNfs21.11’ (×1000); (**E**) cephalic stalk of strain ‘YJNfs21.11’ (×400); (**F**) sporophore of strain ‘YJNfs21.11’ (×400); (**G**) conidiophores of strain ‘YJNfs21.1’ under scanning electron microscope (×5500); (**H**) sporophore of strain ‘YJNfs21.1’ under scanning electron microscope (×1000); (**I**) conidial and mycelial morphology of strain ‘YJNfs21.11’ under scanning electron microscope (×700).

After 24 h of culture on PDA, the strain formed loose, flat, regular-edged colonies 2–3 cm in diameter (Figure 1A). The colonies were initially white with short, fluffy hyphae growing outward (Figure 1B). The center of the colonies later turned green, with a large number of green spores growing (Figure 1C,D). The nutritional hyphae exhibited septa, and a portion of the aerial hyphae formed a long, rough conidium, giving rise to a nearly spherical apical sac (Figure 1E). The surface gave rise to several small peduncles bearing clusters of coarse-surfaced spherical conidia (Figure 1F). Additionally, rough-surfaced spherical conidia were generated on the surface of the small peduncle (Figure 1G–I).

**Figure 2:**
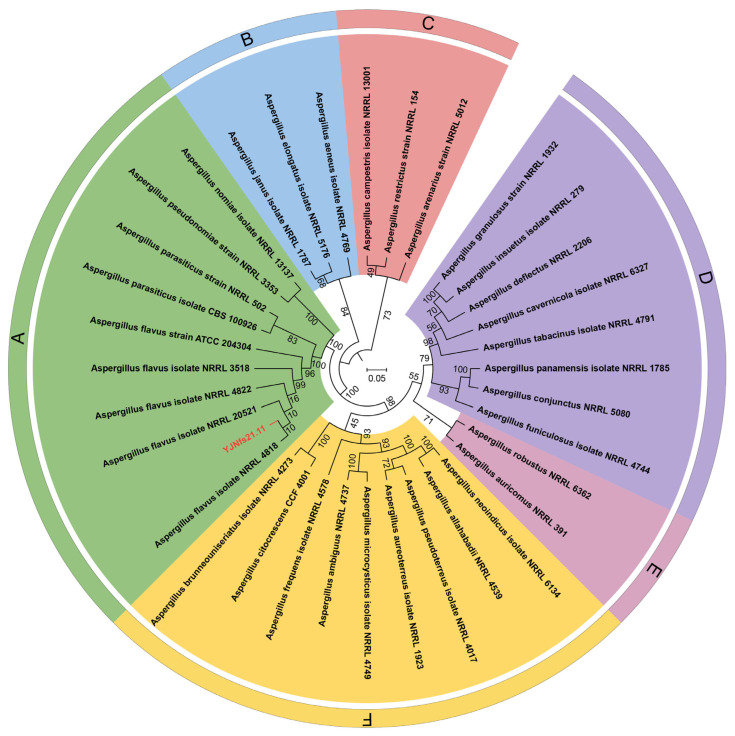
Phylogenetic tree based on concatenated ITS, LSU (28S), and *BenA* of strain ‘YJNfs21.11’. Note: (**A**–**F**) represent different branches of the phylogenetic tree.

Through an ITS gene sequence analysis and search in the BLAST database (NCBI), the strain was identified as *Aspergillus flavus* (GenBank: OP071264), and the homology reached 99.33%. Then, we further analyzed the gene sequences of LSU (28S) and *BenA*, and compared them with the sequences reported in the BLAST database (NCBI). We speculated that this might be a new strain, and we temporarily named it ‘YJNfs11’ for the convenience of subsequent studies. A sample specimen was deposited in the China General Microbiological Culture Collection Center (CGMCC) with the deposit number 40260. It will be used as a new portal for further research, such as whole-genome sequencing analysis. Phylogenetic tree based on concatenated ITS, LSU (28S), and *BenA* of strain ‘YJNfs21.11’ was shown in Figure 2.

### 3.2. Biocontrol Efficacy of A. flavus 

**Figure 3:**
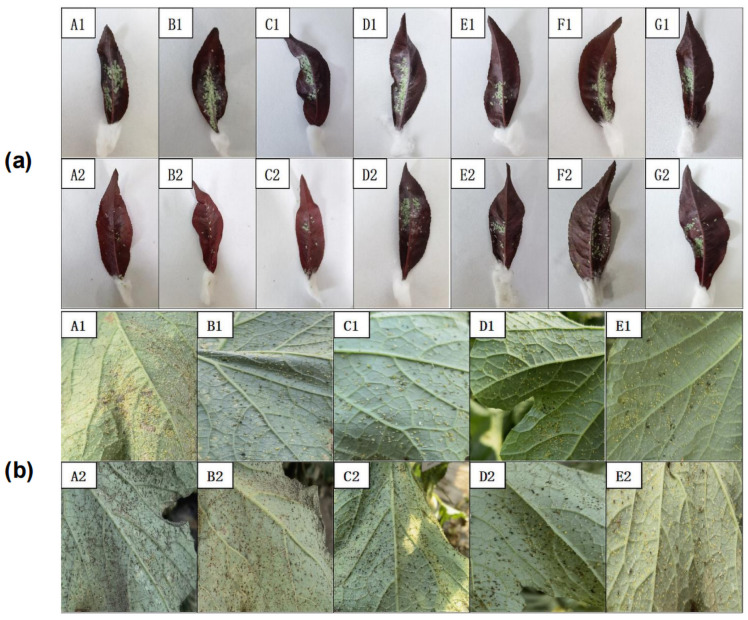
Comparison of the efficacy of each reagent against *H. arundimis* and *A. gossypii* before and after application. Note: (**a**): A1: efficacy of 100× diluted spore suspension 1 h after application; A2: efficacy of 100× diluted spore suspension 48 h after application; B1: efficacy of 200× diluted spore suspension 1 h after application; B2: efficacy of 200× diluted spore suspension 48 h after application; C1: efficacy of 300× diluted spore suspension 1 h after application; C2: efficacy of 300× diluted spore suspension 48 h after application; D1: efficacy of 100× diluted mycelium reagent 1 h after application; D2: efficacy of 100× diluted mycelium reagent 48 h after application; E1: efficacy of methanol extract reagent 1 h after application; E2: efficacy of methanol extract reagent 48 h after application; F1: efficacy of fermentation filtrate 1 h after application; F2: efficacy of fermentation filtrate 48 h after application; G1: efficacy of 0.1% tween20 1 h after application; G2: efficacy of 0.1% tween20 48 h after application. (**b**): A1: efficacy of stock solution 1 d after application; A2: efficacy of stock solution 5 d after application; B1: efficacy of 2× diluted stock solution 1 d after application; B2: efficacy of 2× diluted stock solution 5 d after application; C1: efficacy of 10× diluted stock solution 1 d after application; C2: efficacy of 10× diluted stock solution 5 d after application; D1: efficacy of 50× diluted stock solution 1 d after application; D2: efficacy of 50× diluted stock solution 5 d after application; E1: efficacy of 0.1% tween20 1 d after application; E2: efficacy of 0.1% tween20 5 d after application.

For laboratory experiments, the corrected control efficacy of the 100× diluted spore suspension reached 85.69% after 48 h (Table 1). These results suggest that the best infective agents were spores. The biocontrol efficacy of each solution, before and after application, is shown in Figure 3a.

Field experiments revealed that *A. flavus* ‘YJNfs21.11’ and its fermentation products exerted considerable control on aphids, with a corrected control efficacy of 96.87% after 5 days of treatment with the undiluted fermentation liquid (Table 2). The biocontrol efficacy of each solution, before and after application, is shown in Figure 3b.

### 3.3. Histopathological Changes of Infected Aphids

#### 3.3.1. External Symptoms of Aphids Infected with *A. flavus* ‘YJNfs21.11’

**Figure 4:**
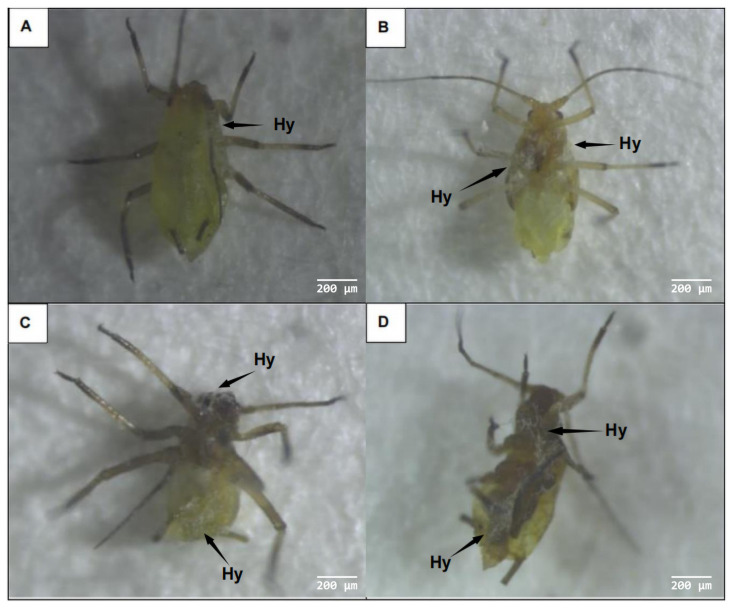
External symptoms of aphids infected with *A. flavus* ‘YJNfs21.11’. Note: (**A**) aphids infected with strain ‘YJNfs21.11’ for 24 h exhibited no change in body color; (**B**) aphids infected with strain ‘YJNfs21.11’ for 36 h, with mycelium attached to the aphid’s abdomen resulting in a darker color; (**C**) aphids infected with strain ‘YJNfs21.11’ for 48 h exhibited significantly decreased mobility and stiffened limbs; (**D**) aphids infected with strain ‘YJNfs21.11’ for 60 h, with mycelium attached to the aphid’s head, abdomen, and tail, resulting in shrinkage.

Observation under a stereomicroscope revealed no significant changes in the mobility or physical signs of aphids between 24 h and 36 h after infection (Figure 4A,B). However, aphid mobility decreased considerably after 48 h of infection, with aphids exhibiting a darkened body color and black intersegmental folds (Figure 4C). After 60 h of infection, white hyphae were observed on the heads, abdomens, and tails of aphids (Figure 4D), indicating that they had died [36]. Furthermore, their bodies had shrunk and stiffened. Aphids infected via spore transmission can form new sources of infection that continue to invade other aphids [37].

#### 3.3.2. Adhesion and Germination of *A. flavus* ‘YJNfs21.11’ on the Surface of Aphids

**Figure 5:**
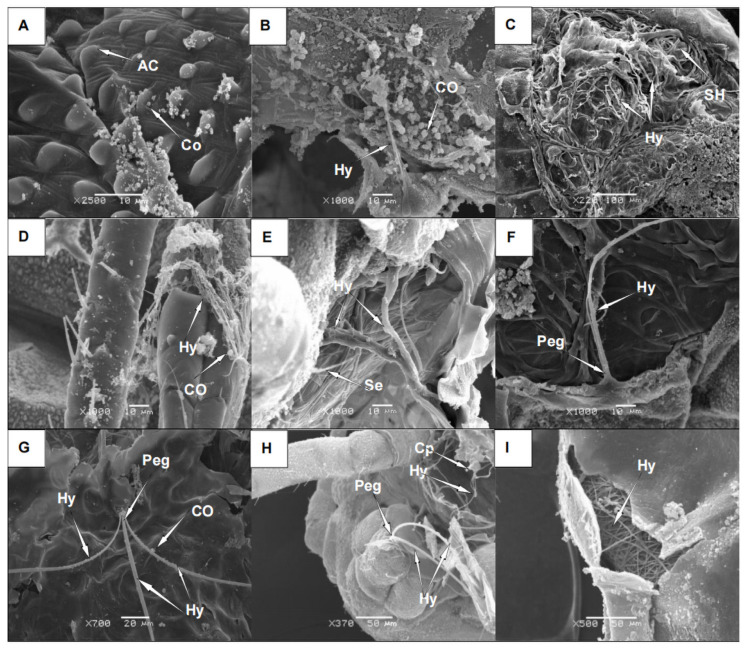
Attachment and germination of *A. flavus* ‘YJNfs21.11’ on aphid body surface. Note: (**A**) conidia (Co) attach to the aphid near acanthoid processes (AC); (**B**) growth and extension of germ tubes to form mycelium (Hy); (**C**) mycelium (Hy) attaches to the surface of the aphid near scaly hairs (SH); (**D**) portion of mycelium (Hy) attached to aphid legs; (**E**) mycelium (Hy) searches for invasion sites near setae (Se); (**F**) the mycelial tip (Hy) at the abdominal internode fold is specialized into an invasive nail (Peg); (**G**) mycelium (Hy) invades the abdominal aphid surface; (**H**) mycelium (Hy) and sporophore (Cp) invade aphid tail; (**I**) cracks and voids appear in the aphid body wall and mycelium was observed inside the aphid body.

Scanning electron microscopy revealed that *A. flavus* ‘YJNfs21.11’ primarily invaded aphid bodies by penetrating the epidermis. After 24 h of infection, the conidia were observed to be attached to the head vertices, antennae, and bases of the mouthparts. The conidia further attached to the spinous projections and intersegmental folds on the thorax and abdomen (Figure 5A,B). After 48 h of infection, the conidia gradually germinated to form germ tubes specialized in appressoria and penetration pegs at the invasion site (Figure 5C,D). The conidia penetrated the epidermis using mechanical force, causing cracks, blackening the aphid body surface, and deforming the epidermis (Figure 5E). During the invasion process, the epidermal tissue was destroyed and cracks and cavities appeared, likely because the fungus secreted enzymes on the surface of the insect (Figure 5F). Many spores penetrated the body wall through these cracks, thereby completing the invasion process (Figure 5G). Through examination of magnified photographs, we observed that the mycelium had many conidia attached to it (Supplementary Materials). We also examined photographs of aphid bristles and, through comparison, we identified the structure as mycelium rather than bristles. The mucilage produced by these spores promoted spore adhesion, germination, and aphid invasion (Figure 5H). Some of the hyphae grew and extended around the setae (Figure 5E). Fungal growth in the peripheral space of the anterior epidermis is generally lateral and can rupture the epidermis, thereby facilitating invasion [38]. At the invasion site, the body surface appeared concave, and the body wall appeared darker in color. In summary, fungal invasion destroyed the epidermal layer and resulted in cracks forming on the surface of the aphid body, allowing *A. flavus* ‘YJNfs21.11’ to enter (Figure 5I).

#### 3.3.3. Invasion of the Aphid Internal Organs by *A. flavus* ‘YJNfs21.11’

**Figure 6:**
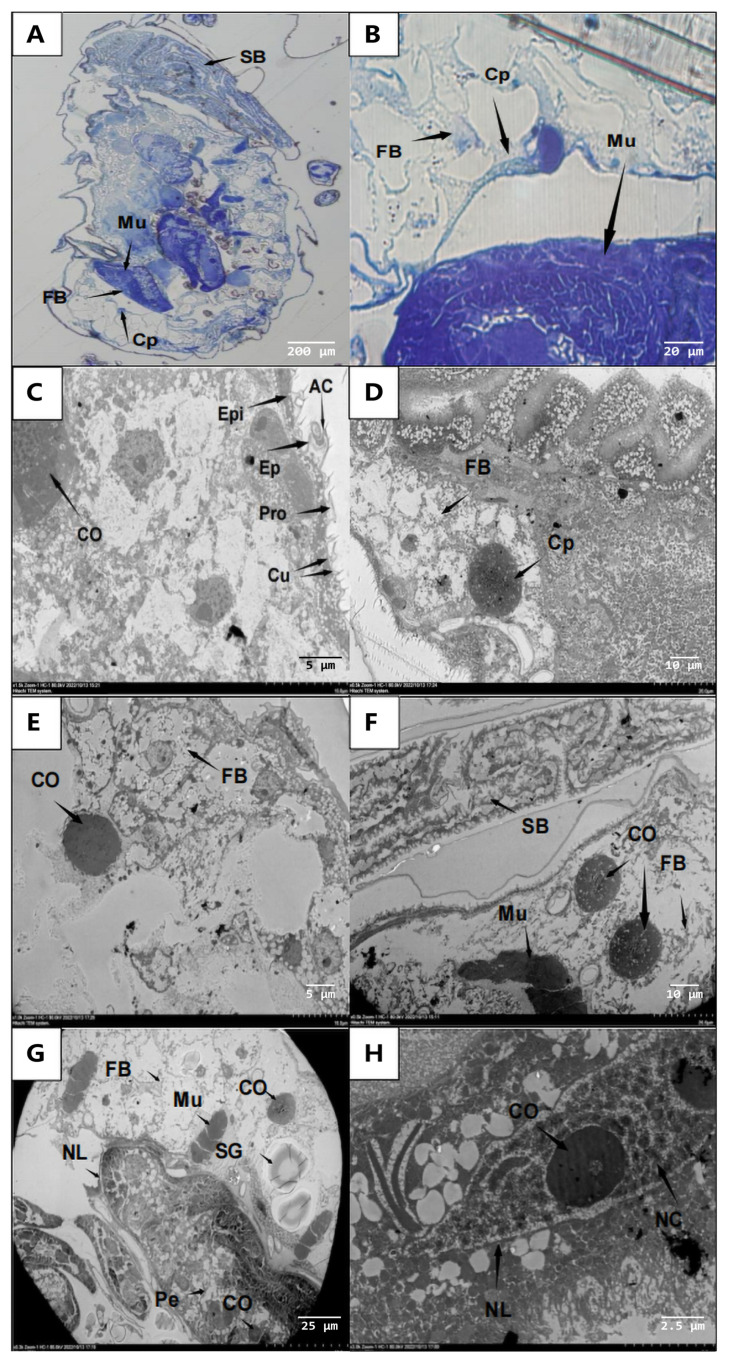
Invasion of aphid fatty layer, muscle, and sheath cell layer by *A. flavus* ‘YJNfs21.11’. Note: (**A**) relatively complete aphid body structure as observed under ultrathin section; (**B**) sporophores (Cp) were observed in the fatty layer; (**C**) from outside to inside, the aphid epidermis consists of spinous process (AC), upper epidermis (Ep), proto-epidermis (Pro), epidermis (Cu), and dermis (Epi); (**D**) sporophore (Cp) observed in aphid body; (**E**) conidia (Co) destroy the fat structure (FB) in vivo; (**F**) muscle (Mu) is loosely divided and small intestine (SB) is relatively intact; (**G**) conidia (Co) enter the sheath cell layer (Pe) through the nerve perinema (NL); (**H**) the nerve cell body (NC) is dissolved and blackened, although the structure of the nerve perimembrane (NL) is relatively intact.

Tissue sections and transmission electron microscopy revealed that the overall aphid body structure was intact after 24 h of infection (Figure 6A,B). From the outside to the inside, the aphid body wall consists of the upper epidermis, pro-epidermis, and dermis (Figure 6C). Muscles and fat are distributed in the hemocoel between the digestive tract and body wall. The muscles are attached to the inner surface of the body wall and the surface of the digestive tract in a strip or block shape. In addition, body fat is linearly distributed in the hemocoel (Figure 6D). After 48 h of infection, the spores had invaded the aphid body, resulting in the loosening of fat structures, the shredding of muscle tissues, and the infection of nerve ganglia (Figure 6E,F). In addition, the nerve cell bodies appeared slightly blackened and some had dissolved (Figure 6G,H). In the late stages of infection, fat was consumed by the spores and the tissue became unstructured. 

### 3.4. Effect of Strain YJNfs21.11 on Extracellular Enzyme Activity during Aphid Body Wall Degradation

**Figure 7:**
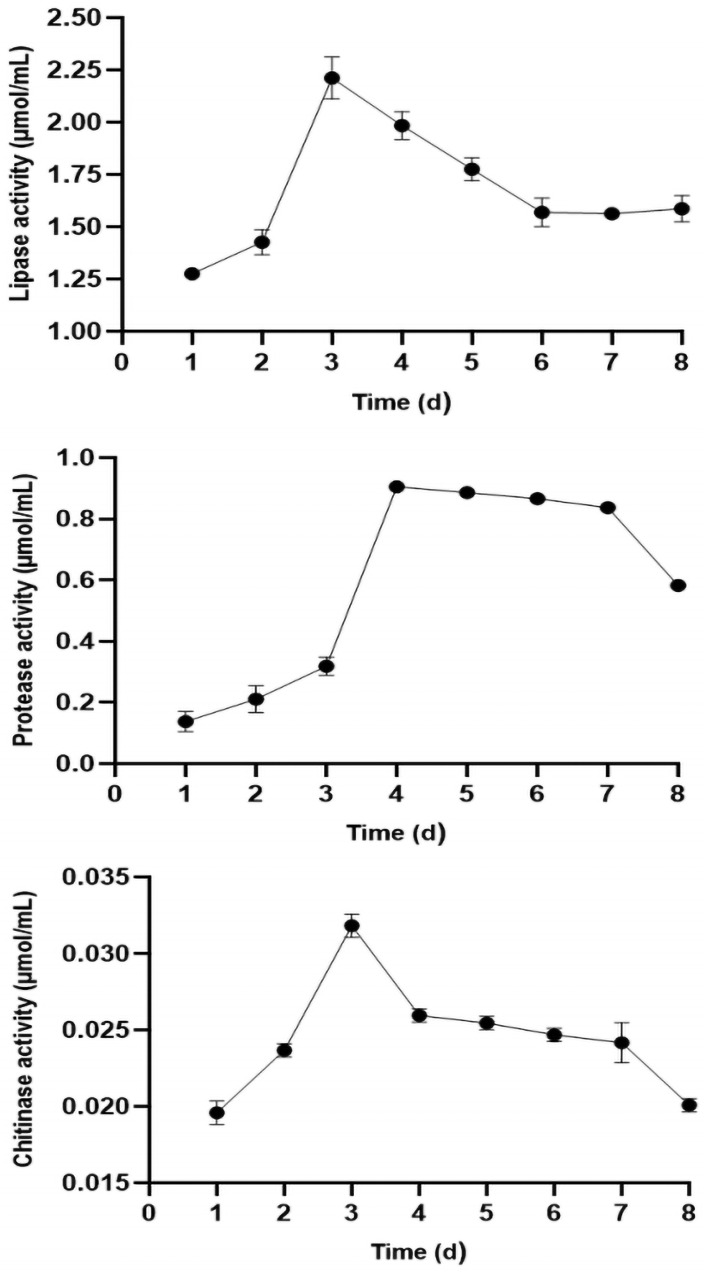
Enzymatic activity over time.

During the degradation of the *H. arundimis* body wall, the activities of lipase, protease, and chitinase tended to first increase and then decrease over time (Figure 7). Both lipase activity (2.212 U/mL) and chitinase activity (0.032 U/mL) were highest on the 3rd day. Protease activity peaked last, and its activity (0.906 U/mL) was highest on the 4th day. During days 3–6, the body wall cracked, the aphid body stiffened and atrophied, and many tissues and organs collapsed, resulting in a decline in lipase activity. In terms of absorbance, the content of the lipase-specific cleaved substrate was significantly higher than that of chitinase or protease. Taken together, these results indicate that lipase played an important role in the degradation of the aphid body wall.

### 3.5. Relationship between Extracellular Enzyme Activity and A. flavus Pathogenesis

Linear regression was performed between enzymatic activity and corrected mortality rates on days 1, 3, and 5 of infection (Table 3). The regression curve of corrected efficacy and lipase activity was y = 36.254x − 1.7864 and the correlation coefficient (R^2^) was 0.7191. The regression curve of corrected efficacy and protease activity was y = 98.036x + 12.999 and R^2^ = 0.8707. The regression curve of corrected efficacy and chitinase activity was y = 2433x − 0.92 and R^2^ = 0.6681. In conclusion, the activities of lipase, protease, and chitinase were positively correlated with aphid mortality. These results suggest that during the late stage of infection, cracks appearing in the aphid body wall were the result of enzymatic breakdown. In this study, the effects of extracellular enzymes on aphid body wall degradation and their virulence were investigated, which provides a theoretical basis for the application of *A. flavus* for the biological control of aphids.

## 4. Discussion

Certain fungi penetrate the host body through a combination of mechanical pressure and enzymatic degradation [39,40]. After germination, spores form germ tubes and appressoria under suitable conditions [41,42]. Each appressorium produces only one penetration peg which exerts a selective effect on nutrients before invading the body, resulting in physical pressure and forcing the penetration peg to invade the nutrient-rich aphid body [43,44].

Using transmission electron microscopy, we observed considerable degradation of the aphid epidermis around the infection site [45]. In fact, conidial germ tubes are known to secrete substances that degrade extracellular enzymes, decompose or soften the insect body wall [46], and aid in the invasion of the insect body [47]. These enzymes, such as esterase, protease, and chitinase, can also provide nutrients required for fungal growth and reproduction [48,49]. During the late stages of infection, a portion of the upper aphid epidermis disappeared, indicating that *A. flavus* spores may secrete extracellular lipases, proteases, and chitinases during invasion. This was later confirmed when lipases activity was found to reach the maximum on the 3rd day.

To date, the majority of research on fungal biocontrol agents has focused on simple insecticidal assays [50,51]. These simple experiments do not address the process of fungal infection nor the mechanism by which fungi exert control on their insect hosts, and generally do not study the enzymatic digestion process in detail. In this study, the mechanism by which *A. flavus* ‘YJNfs21.11’ infects aphids was studied in depth, including the enzymatic dynamics associated with the infection process. Because spores were identified as the infective agent, our study focused on spores. However, we cannot rule out that either the mycelia or other bioactive metabolites may be involved in the process, and suggest further study.

*Aspergillus flavus* is distributed all over the world, especially in warm and moist fields [52]. It can produce a variety of secondary metabolites [53], among which aflatoxins have been most studied because of their strong carcinogenicity [54], which includes AFB1, AFB2, AFG1, and AFG2 [55]. *A. flavus* strains AF13 and K54A are notorious for producing aflatoxins [56]. However, not all A. flavus produce aflatoxin [57,58,59,60]; for example, NRRL 21882 [61] and SU-16 [62,63] are non-toxin-producing strains and have been used for biological control of plant diseases and brewing of yellow rice wine, respectively [64]. In this study, the fermentation products of *A. flavus* ‘YJNfs21.11’ were detected with LC-MS (AB SCIEX, Singapore), and no aflatoxins were detected (Supplementary Materials). Meanwhile, we also did not detect the presence of aflatoxin synthesis-related genes using PCR (Supplementary Materials). *A. flavus* ‘YJNfs21.11’ was isolated and extracted from soil, and its metabolites were natural products; these natural products can be degraded and reused by microorganisms. In field and laboratory bioassays, the study was carried out between biological half-lives [65]. Therefore, *A. flavus* ‘YJNfs21.11’ is safe for the environment and people. In addition, strategies for the degradation of other aflatoxin-producing strains of aflatoxin are now available. One major determinant of aflatoxin production in *A. flavus* is temperature, and oxidative stress may also indeed be a pre-requisite for aflatoxin production [66,67]. The most promising strategy currently being used to reduce preharvest contamination of crops with aflatoxin is to introduce non-aflatoxin (biocontrol) *A. flavus* into the crop environment [68,69]. In conclusion, *A. flavus* is worth exploring as a promising biocontrol agent. We believe that this work will provide new ideas and directions for related research.

## 5. Conclusions

*A. flavus* ‘YJNfs21.11’ effectively infects aphids through conidial spores. The infection process results in the destruction of the epidermal tissue and internal organs, and the eventual death and digestion of the aphid. The activities of lipase, protease, and chitinase are positively correlated with aphid mortality, and they complete the infection process by decomposing aphid epidermal auxiliary spores. These findings highlight that biocontrol agents can successfully replace chemical pesticides for aphid control [70,71,72] and *A. flavus* is worth exploring as a promising biocontrol agent [73,74].

## Figures and Tables

**Table 1:** Mortality and corrected efficacy of *A. flavus* ‘YJNfs21.11’ against *H. arundimis*.

Process	16 h		24 h		48 h	
Number	Mortality (%)	Corrected Efficacy (%)	Mortality (%)	Corrected Efficacy (%)	Mortality (%)	Corrected Efficacy (%)
spore suspension diluted 100×	(48.04 ± 6.13) b	(47.385 ± 6.21) b	(62.93 ± 3.87) ab	(54.62 ± 4.74) ab	(91.89 ± 3.07) a	(85.69 ± 5.41) a
spore suspension diluted 200×	(36.78 ± 6.97) bc	(35.99 ± 7.05) bc	(47.30 ± 1.54) bc	(35.49 ± 1.88) bc	(84.56 ± 2.19) abc	(72.76 ± 3.86) abc
spore suspension diluted 300×	(23.11 ± 5.37) cd	(22.15 ± 5.43) cd	(34.03 ± 4.77) cd	(19.24 ± 5.83) cd	(78.21 ± 0.40) bc	(61.57 ± 0.71) bc
mycelium reagent diluted 100×	(31.85 ± 16.27) bc	(31.00 ± 16.48) bc	(44.21 ± 4.16) bc	(41.39 ± 20.42) bc	(76.55 ± 3.94) c	(58.63 ± 6.96) c
methanol extract	(3.98 ± 9.34) de	(2.78 ± 9.45) de	(8.25 ± 17.78) e	(−13.80 ± 21.16) e	(45.66 ± 15.95) d	(4.13 ± 28.13) d
fermentation filtrate	(71.07 ± 9.90) a	(70.71 ± 10.03) a	(82.64 ± 10.12) a	(79.59 ± 13.13) a	(90.62 ± 3.47) ab	(83.46 ± 6.12) ab
negative control	(0.24 ± 18.09) e	(−1.01 ± 18.32) e	(17.37 ± 17.45) de	(−1.16 ± 21.36) de	(36.89 ± 4.35) d	(−11.34 ± 7.67) d
F	F = 14.384	F = 14.384	F = 17.635	F = 14.029	F = 32.053	F = 32.054
	*p* < 0.001	*p* < 0.001	*p* < 0.001	*p* < 0.001	*p* < 0.001	*p* < 0.001

Note: All data are mean ± standard deviation. Different lowercase letters within the same column indicate significant differences between treatments (*p* < 0.05).

**Table 2:** Mortality and corrected efficacy of *A. flavus* ‘YJNfs21.11’ against *A. gossypii*.

Process	1 d		3 d		5 d	
Number	Mortality (%)	Corrected Efficacy (%)	Mortality (%)	Corrected Efficacy (%)	Mortality (%)	Corrected Efficacy (%)
stock solution	(73.63 ± 6.03) a	(75.19 ± 5.68) a	(80.61 ± 1.89) a	(80.58 ± 1.89) a	(98.32 ± 1.01) a	(96.87 ± 1.88) a
stock solution diluted 2×	(68.69 ± 4.15) a	(70.54 ± 3.90) a	(77.61 ± 2.24) ab	(77.58 ± 2.24) ab	(92.51 ± 3.53) ab	(90.30 ± 2.33) ab
stock solution diluted 10×	(60.64 ± 6.18) a	(62.97 ± 5.82) a	(78.30 ± 0.93) ab	(78.27 ± 0.93) ab	(90.70 ± 0.65) b	(82.72 ± 1.20) bc
stock solution diluted 50×	(30.40 ± 9.47) b	(34.22 ± 9.31) b	(72.17 ± 4.78) b	(72.13 ± 4.79) b	(89.50 ± 5.57) b	(75.43 ± 1.68) c
negative control	(−5.05 ± 14.46) c	(1.17 ± 13.60) c	(9.23 ± 7.40) c	(9.10 ± 7.41) c	(49.42 ± 4.27) c	(6.02 ± 7.93) d
F	F = 41.702	F = 40.932	F = 160.826	F = 160.828	F = 91.950	F = 266.98
	*p* < 0.001	*p* < 0.001	*p* < 0.001	*p* < 0.001	*p* < 0.001	*p* < 0.001

Note: All data are mean ± standard deviation. Different lowercase letters within the same column indicate significant differences between treatments (*p* < 0.05).

**Table 3:** Mortality and corrected efficacy of spore suspension against *H. arundimis*.

Process Number	1 d		3 d		5 d	
	Mortality (%)	Corrected Efficacy (%)	Mortality (%)	Corrected Efficacy (%)	Mortality (%)	Corrected Efficacy (%)
Spore suspension	(30.49 ± 2.17)	(26.29 ± 2.30)	(67.76 ± 4.44)	(64.90 ± 4.84)	(92.47 ± 1.75)	(91.26 ± 2.03)
Blank control	(6.81 ± 5.02)	-	(9.52 ± 4.14)	-	(16.24 ± 5.51)	-

## Data Availability

All experimental data are included in this paper and the Supplementary Materials.

## Supplementary Materials

The following supporting information can be downloaded at: https://www.mdpi.com/article/10.3390/microorganisms11112788/s1, Figure S1. LC-MS chromatogram. Figure S2. Aflatoxin synthesis gene was detected using PCR. Figure S3. Attachment and germination of *A. flavus* ‘YJNfs21.11’ on aphid body surface. Table S1. Sequences of the nucleotide primers used in PCR study. Refs. [73,74] can be found in Supplementary Materials.
