# Supplementary material for: Discovery, Identification, and Insecticidal Activity of an Aspergillus flavus Strain Isolated from a Saline–Alkali Soil Sample
Source: Microorganisms. 2023 Nov 16;11(11):2788. doi: 10.3390/microorganisms11112788 (PMC10673062; doi:10.3390/microorganisms11112788)
Supplement: Supplementary file 1 [file microorganisms-11-02788-s001.zip › microorganisms-2684199-supplementary.pdf]

Supplementary Material

Discovery, Identification, and Insecticidal Activity of an *Aspergillus flavus* Strain Isolated from a Saline–Alkali Soil Sample

1. ***Identification of ITS DNA***

CTTTTCGCTCGTGGGGGTACTGCGGAAGGATCATTACCGAGTGTAGGGTTCCTAGCGAGCCCA
ACCTCCCACCCGTGTTTACTGTACCTTAGTTGCTTCGGCGGGCCCGCCATTCATGGCCGCCGGGG
GCTCTCAGCCCCGGGCCCGCGCCCGCCGGAGACACCACGAACTCTGTCTGATCTAGTGAAGTCT
GAGTTGATTGTATCGCAATCAGTTAAAACTTTCAACAATGGATCTCTTGGTTCCGGCATCGATGA
AGAACGCAGCGAAATGCGATAACTAGTGTGAATTGCAGAATTCCGTGAATCATCGAGTCTTTGA
ACGCACATTGCGCCCCCTGGTATTCCGGGGGGCATGCCTGTCCGAGCGTCATTGCTGCCCATCAA
GCACGGCTTGTGTGTTGGGTCGTCGTCCCCTCTCCGGGGGGGACGGGCCCCAAAGGCAGCGGC
GGCACCGCGTCCGATCCTCGAGCGTATGGGGCTTTGTCACCCGCTCTGTAGGCCCGGCCGGCGC
TTGCCGAACGCAAATCAATCTTTTCCAGGTTGACCTCGGATCAGGTAGGGATACCCGCTGAACTT
AAGCATATCAATAAGGGGGAGGAACCCTTGTGGGTGTCTTTAATGGCTTTAACCCCCCCCCCCGC
TCCCTATAGGGGAGATCTTTTCTTCGAACTGGTGTAATTTGCATACGTTGAGTTTTGATCAGTGTG
CCCCCCCCGCACATAAAGGCCCCCAACAACACCTCTAGATACGACGATTTCGGGGGATCCGTGC
TTATATCAAAAAAAAATTCCCTACATTATGTCCCCGTGCTACCTGTAGACCCATTGCATGGACGA
GATACTGAGATAGGGGAAATGTAGAAATCCCCAAGAAATCAACATTATAAGCCATATCCCGGGG
CACCACGCGATCCTTTGTAGCCACGTCCCCCGCCTTCGTCCCCCCCCCAAAAACTGGCGGGGAT
ACTCCCCGCCCGGGGG

2. ***Identification of LSU (28S) DNA***

CTGGCAGGTAGCGTCGCGGCACTGCCTGGTCAGACAGCCGCAAAAACCAATTATCTGAATCAAC
GGTTCCTCTCGTACTAAATTGAATTACCGTTGCGGCGACCTTCATCAGTAGGGTAAAACTAACCT
GTCTCACGACGGTCTAAACCCAGCTCACGTTCCCTATTAGTGGGTGAACAATCCAACGCTTACCG
AATTCTGCTTCGGTATGATAGGAAGAGCCGACATCGAAGGATCAAAAAGCAACGTCGCTATGAA
CGCTTGGCTGCCACAAGCCAGTTATCCCTGTGGTAACTTTTCTGGCACCTCTAGCCTCAAATTTCG
AGGGACTAAAGGATCGATAGGCCACACTTTCATGGTTTGTATTCACACTGAAAATCAAAATCAA
GGGGACTTTTACCCTTTTGTTCTACTGGAGATTTCTGTTCTCCATGAGTCCCCCTTAGGACACCTG
CGTTGTGGTTTAACAGATGTGCCGCCCCAGCCAAACTCCCCACCTGACAATGTCTTCAACCCGG
ATCGGCCCGCGAAGGACCTTAACGCCAGAAGATGGGCGGTGAAGCCCAGTTCCGCTTCATTGA
ATAAGTAAAAAAACGATAAAGGTAGTGGTATTTCACTGGCGCCGGAGCTCCCACCTATTCTACAC
CCCATATGTCTTTTCACAATGTCAAACTAGAGTCAAGCTCAACAGGGTCTTCTTTCCCCGCTGATT
CTGCCAAGCCCGTTCCCTTGGCTGTGGTTTCGCTAGATAGTAGATAGGGACAGTGGGAATCTCGT
TAATCCATTCATGCGCGTCACTAATTAGATGACGAGGCATTTGGCTACCTTAAGAGAGTCATAGTT
ACTCCCGCCGTTTACCCGCGCTTGGTTGAATTTCTTCACTTTGACATTCAGAGCACTGGGCAGAA
ATCACATTGCGTCAACACCACTTTCTGGCCATCGCAATGCTATGTTTTAATTAGACAGTCAGATTC
CCCTTGTCCGTACCAGTTCTAAGTTGGTCGTTAAGCGCCCGCCGGACGGCCGAAGCCTGCCAAG

GGCGTCCCCCACCCGGTGCTGGGGGGCGCCGGCCGGTTGCCCGGCTAGGGCCCCCCTGCTCCGA
GGGGGTTCCCCCAAGGGCCACGGAGGCCCGACCCTTTAGAGCCAATCCTTAATCCCGAAGTTAC
GGGATCCATTTTGGCCAACTTCCCTTAACAACATTGTTCA

3. *Identification of beta-tubulin*

TGGTAACCAAAATCGGTGCTGCTTTCTGGTATGTCTCAATGCCTTCGAGTTAGTATGCTTTGGACC
AAGGAACTCCTCAAAAGCATGATCTCGGATGTGTCCTGTTATATCTGCCACATGTTTGCTAACAA
CTTTGCAGGCAAACCATCTCTGGCGAGCACGGCCTTGACGGCTCCGGTGTGTAAGTACAGCCTG
TATACACCTCGAACGAACGACGACCATATGGCATTAGAAGTTGGAATGGATCTGACGGCAAGGA
TAGTTACAATGGCTCCTCCGATCTCCAGCTGGAGCGTATGAACGTCTACTTCAACGAGGTGCGTA
CCTCAAAATTTCAGCATCTATGAAAACGCTTTGCAACTCCTGACCGCTTCTCCAGGCCAGCGGA
AACAAGTATGTCCCTCGTGCCGTCCTCGTTGATCTTGAGCCTGGTACCATGGACGCCGTCCGTGC
CGGTCCCTTCGGTCAGCTCTTCCGTCCCGACAACTTCGTTTTCGGCCAGTCCGGTGCTGGTAACA
ACTGGGCCAAGGGTCACTAACCTTGAGGGTA

4. Ultra-High Performance Liquid Chromatography-Mass Spectrometry

4.1. Preparation of fermentation solution

The fermentation medium (35 g soluble starch, 15 g sucrose, 12.5 g yeast extract, 7.5 g soybean cake powder, 1.0 g KH_2PO_4, 1.1 g anhydrous $MgSO_4$, 1.0 g NaCl) was prepared in 1 L of distilled water and distributed into ten 250 mL conical flasks. The flasks were sealed with eight layers of gauze and then autoclaved for 20 min. The strains cultured on the PDA plate were cut from the edge of the colony using a sterilization hole punch with a diameter of 4 mm under sterile conditions, and inoculated into the fermentation medium. Following inoculation, the flasks were again sealed with eight layers of gauze and cultured for 3 d in a shaker (165 r/min) at 32 °C.

4.2. Sample preparation and extraction

Remove the fermentation solution from a shaker and vortex for 30 s to mix. Take an appropriate amount of liquid sample, place it in a corresponding numbered 50mL centrifuge tube, freeze it overnight at -80 °C in the refrigerator, and vacuum freeze dry. Weigh 50 mg of the sample using an electronic balance (MS105DM). The sample were added to 20 mL petroleum ether in 50 mL centrifuge tubes and extracted with ultrasonic waves for 30 min. The products were shaken for three hours at 180 rpm. The supernatant was collected into a flask and dried using a rotary evaporator, and the above extraction steps were repeated three times. Next, 2 mL of liquid were transferred into a 2 mL centrifuge tube, evaporated, then dissolved with 2 mL of chromatographic grade methanol. Filter the sample with a microporous filter membrane (0.22 μm pore size), and save it in the injection vial for UPLC-MS analysis.

4.3. UPLC conditions

The fermentation solution sample extracts were analyzed using an UPLC-ESI-MS system and Tandem mass spectrometry system. The analytical conditions were as follows, UPLC: column, Agilent SB-C18 (1.8 μm, 2.1 mm × 100 mm); The mobile phase was consisted of solvent A, pure water with 0.1% formic acid, and solvent B, acetonitrile with 0.1% formic acid. Sample measurements were performed with a gradient program that employed the starting conditions of 95% A, 5% B. Within 9 min, a linear gradient to 5% A, 95% B was programmed, and a composition of 5% A, 95% B was kept for 1 min. Subsequently, a composition of 95% A, 5.0% B was adjusted within 1.1 min and kept for 2.9 min. The flow velocity was set as 0.35 mL per minute; The column oven was set to 40°C; The injection volume was 2 μL.

4.4. ESI-Q TRAP-MS/MS

The ESI source operation parameters were as follows: source temperature 500°C; ion spray voltage (IS) 5500 V (positive ion mode)/-4500 V (negative ion mode); ion source gas I (GSI), gas II(GSII), curtain gas (CUR) were set at 50, 60, and 25 psi, respectively; the collision-activated dissociation (CAD) was high.

4.5. Result analysis

Aspergillus flavus is a diverse assemblage of strains that include aflatoxin-producing and non-toxigenic strains. In this study, we tested the most critical aflatoxins, such as AFB1, AFB2, AFG1, AFG2, AFM1 and AFGM. We did not find aflatoxins produced by this strain after test of UPLC-MS (AB SCIEX, Singapore). The test results showed that the ion peak of the standard mixed solution was about 200,000, while our test result was about 3000, with a considerably low response value and no peak, indicated that the strain did not produce related aflatoxin.

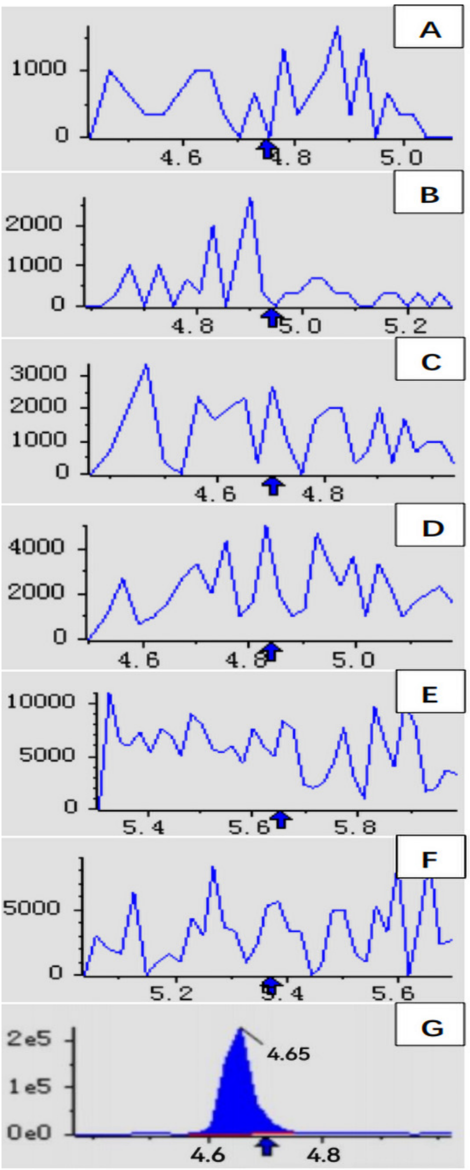

Figure S1. LC-MS chromatogram

Notes: A: Chromatogram of aflatoxin AFB1 detection; B: Chromatogram of aflatoxin AFB2 detection; C: Chromatogram of aflatoxin AFG1 detection; D: Chromatogram of aflatoxin AFG2 detection; E:

Chromatogram of aflatoxin AFM1 detection; F: Chromatogram of aflatoxin AFGM detection; G: Mixed standard solution chromatogram.

5. ***Aflatoxin synthesis gene was detected by PCR***

5.1. primers used in PCR study

The genomic DNA of Aspergillus flavus were detected for the presence of nine key aflatoxin synthesis related genes (*aflP, aflQ, aflS, alfO, alfD, alfM, omtA, AflR* and *aflJ*) by PCR using previously reported primers. The genes, their primer sequences and their product sizes (Table S1) were selected from already reported data [73,74].

Table S1. Sequences of the nucleotide primers used in PCR study.

Number	Target gene	Primer code	Primer sequences	Product size (bp)
1	*aflP*	Omt1-F	GTGGACGGACCTAGTCCGACATCC	870bp
		Omt1-R	GTCGGCGCCACGCACTGGGTTGGG	
2	*aflQ*	Ord-gF	TTAAGGCAGCGGAATACAAG	757bp
		Ord-gR	GACGCCCAAAGCCGAACACAAA	
3	*aflS*	1For	TGAATCCGTACCCTTTGAGG	684bp
		2rev	GGAATGGGATGGAGATGAGA	
4	*aflO*	1For	TCCAGAACAGACGATGTGG	790bp
		2rev	CGTTGGCTAGAGTTTGAGG	
5	*aflD*	Nor1	ACCGCTACGCCGGCACTCTCGGCAC	400bp
		Nor2	GTTGGCCGCCAGCTTCGACACTCCG	
6	*aflM*	Ver1	GCCGCAGGCCGCGGAGAAAGTGGT	537bp
		Ver2	GGGGATATACTCCCGCGACACAGCC	
7	*omtA*	Omt1	GTGGACGGACCTAGTCCGACATCAC	797bp
		Omt2	GTCGGCGCCACGCACTGGGTTGGGG	
8	*aflR*	AflR1	TATCTCCCCCCGGGCATCTCCCGG	1032bp
		AflR2	CCGTCAGACAGCCACTGGACACGG	
9	*aflJ*	AflJF	TGAATCCGTACCCTTTGAGG	737bp
		AflJR	GGAATGGGATGGAGATGAGA	
10	*ITS*	ITS1	TCCGTAGGTGAACCTGCGG	500-800bp
		ITS4	TCCTCCGCTTATTGATATGC	

5.2. The PCR results

As shown in the figure S2, lanes 1, 3, 4, 5, 7, 8, and 9 did not amplify relevant gene bands, and lanes 2 and 6 amplified gene bands, and ITS amplification was normal. We sequenced the genes amplified from lane 2 and lane 6. According to BLAST database (NCBI) search and comparison, their product sizes were 340bp and 295bp respectively, which did not match the two aflatoxin-producing genes.

In conclusion, *Aspergillus flavus* 'YJNfs21.11' does not produce the above nine aflatoxin-producing genes. So it can be inferred that *Aspergillus flavus* 'YJNfs21.11' does not produce related aflatoxins.

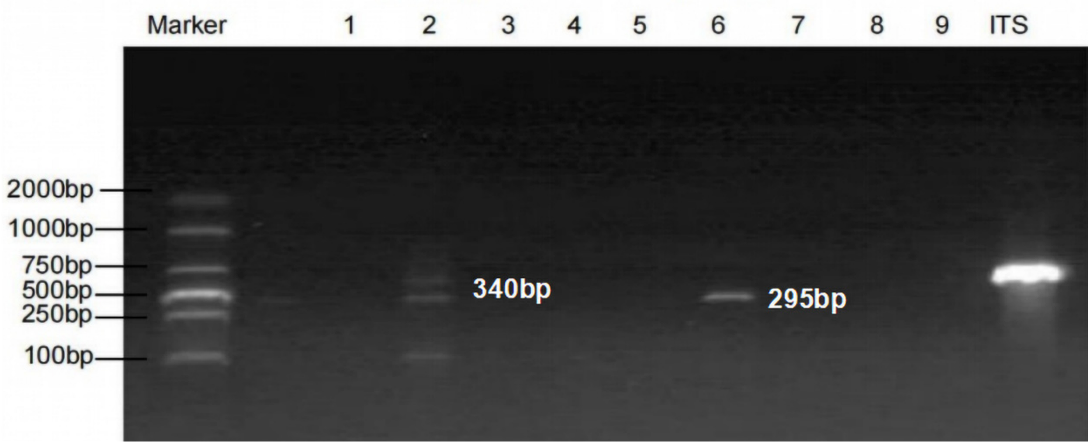

Figure S2. Aflatoxin synthesis gene was detected using PCR

Notes: amplifcation of afatoxin biosynthetic genes *aflP* (1), *aflQ* (2), *aflS* (3), *aflO* (4), *aflD* (5), *aflM* (6), *omtA* (7), *aflR* (8), *aflJ* (9) and ITS; Marker: GL 2000.

5.2.1. Sequence of band in lane 2

GCGGGCCGAAGATGCACGGGGAAGGAACGCAGGAGTCAACTCAGGGTGGAGGACATTCGGAC
ATGACTTTGGTCATCGCCTTCTCCGTTTACCACCCGCTTATTCCCTAAGGTGATTGACCACCACCG
TTGATTCCTTACTGCATCTCGGAACAATCAATACCCCGACTGCTACTGGTCTCTCTACCCTCGTCG
TGGTCGTTCCTATCTCATTACCTGGGCCCTAACTGCGACCGTACCCACCCGACCTCCTGTAATGTC
CTCCACCCTGAGCTTCACCTCTGACAATTTGCACCATGATACCTTCTCCGACCATGTGTTTCTCCG
AGGATCTGCCCAAA

5.2.2 Sequence of band in lane 6

ATGGTTCCGTACTTATCTGATAAGGAACAGGTCGCTCGCGGCTGCTCTCGTCCTTCCATCCTCTTG
CGCTTGTTGCAGAAAGGGCACTATGAATCTGGATCACATCAAGAGCAGGTTTTGGAATGGACTA
ATGCCGAGTTCGTTATGGGAGGATCGGACACGGAACAACATTCTCTGCCTCATCATTTTCATATA
AGAAGCGATCCTCTCGCTTGCTCTGACGGGGTCCGCAAGGGCAGGCTTGATGCCGTCCATGCAC
CTGCTTGACGAGGGCGTCCGCAAGGCGCGCGAGGAA

6. *Evaluation of histopathologic changes in infected aphids*

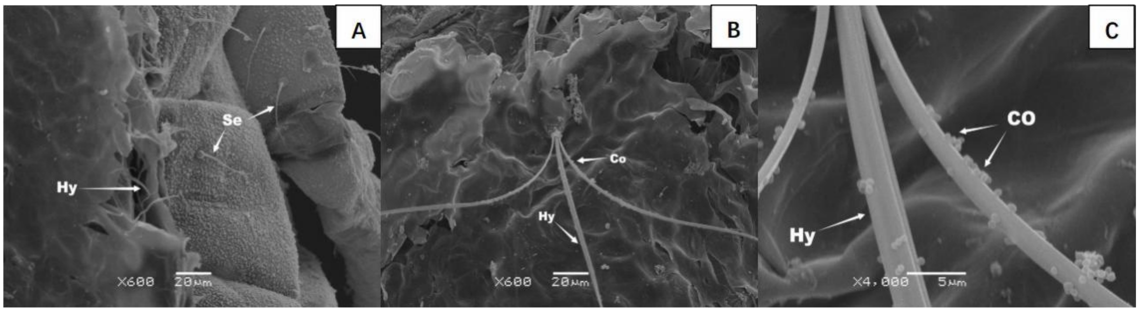

Figure S3. Attachment and germination of *A. flavus* 'YJNfs21.11' on aphid body surface

Notes: A: setae of aphid by scanning electron microscope (×600); B: mycelium (Hy) invades the abdominal aphid surface by scanning electron microscope (×600); C: conidia (Co) attach to aphid near mycelium (Hy) by scanning electron microscope (×4000).
